# “Neonatal seizure: how reliable is its diagnosis and treatment? A mini review of previous knowledge”

**Published:** 2020

**Authors:** Mahmoud MOHAMMADI, Zahra REZAEI

**Affiliations:** 1 Head Department of Pediatric Neurology, Clinical Neurophysiology and Epilepsy, Children’s Medical Center, Pediatrics Center of Excellence, Tehran University of Medical Sciences, Tehran, Iran.; 2 Child Neurologist, Neurology Clinic, Shahid Fahmideh Children Hospital, Iran University of Medical Sciences, Tehran, Iran.

**Keywords:** Neonatal seizure, Amplitude integrated electro encephalogram, Anti-seizure medication

## Abstract

Seizure is the most common neurologic event in the neonatal period when the immature, growing brain is vulnerable to various injuries. Seizure might be present as an atypical feature in neonates, which makes diagnosis a challenge. A vast majority of seizures are symptomatic due to an underlying cause, searching for etiology to remove them leads to more effective therapy. However, there is doubt about the diagnosis of seizures and the best treatment for them.

Here, we reviewed articles related to diagnosis and treatment of neonatal seizures to evaluate the available evidence.

The results showed that despite numerous studies on the topic, neither an implicit diagnosing method nor a therapeutic regime was proposed. It was also observed that phenobarbital prescription was reduced while newer anti-seizure medication such as levetiracetam was further prescribed.

Seizure, the most common neurologic disorder in neonates, is a challenging topic for both neonatologists and neurologists. Since patients are critically ill, designing a randomized clinical trial appears not to be easy for neonates admitted to a neonatal intensive care unit. Moreover, both the diagnosis and treatment of seizures should be re-evaluated based on neonatal characteristics. In the recent decade, new less harmful anti-seizure medications are being replaced with old ones.

## Introduction

Seizure is the most common neurologic manifestation in the neonatal period (1). Seizure occurs in five out of every 1000 term neonates, and is even more frequent among preterm neonates (2). The prevalence of neonatal seizure appears to be more in low-income countries than in higher-income ones, although there is limited data (3). 

Here, we reviewed the previous research to evaluate how reliable the data was concerning neonatal seizure. 

The immature brain is more vulnerable to seizure due to several factors such as primary development of excitatory neurotransmitters, delayed inhibition of GABA neurotransmitters, and increase of excitatory glutaminergic neurons (2). The developing brain is considered as a great topic for debate: “how vulnerable or resistant is it to injuries such as seizures”. In most cases, an acute event leads to neonatal seizure, including Hypoxic Ischemic Encephalopathy (HIE), the central nervous system (CNS) infection, electrolyte imbalance, hemorrhage, cerebral dysgenesia, and in rare cases, neonatal epileptic syndromes (4). Thus, prompt evaluation to treat the underlying cause is essential. On the other hand, since the cause is acute, prolonged treatment appears to be unnecessary (5).

Neonatal seizures have neither classic clinical seizure manifestations nor electrographic counterparts. In other words, the electro clinical dissociation and incongruity between clinical and electrical features are observed in many neonatal seizure cases, which make diagnosis challenging. Diagnosis only based on clinical manifestations is not justified because of atypical, subclinical, and pure electrical seizures (6). Subtle presentation could be the sole seizure manifestation in neonates (i.e., eye deviation, staring, chewing, sucking, cycling or boxing limb movements, apnea, and blood pressure changes) (7).

Ictal neonatal electroencephalogram (EEG) is characterized by any rhythmic, repetitive, stereotypic discharges lasting for more than 10 seconds in at least two channels, with delta/theta frequencies being the most common (6,8 ).

EEG is needed in both seizure diagnosis and treatment efficacy monitoring (6). EEG monitoring in neonatal seizures is obligatory for both diagnosis and treatment monitoring is obligatory not optional. Several studies suggest that neonatal seizures could be considered as a biomarker for brain function and not only as a disease. This is another major feature of neonatal electrographic seizure evolution that could be observed in frequency, morphology, and voltage (9).

EEG video monitoring is gold standard for detection of neonatal seizures. However, it is not easily available commercially and there is a limited number of trained operators and interpreters to implement it (9). Amplitude integrated EEG (aEEG) is another technique which has been used since 1990 in the neonatal intensive care unit (NICU) (10) as a useful screening method for brain function monitoring (11). Brain signals are amplified and pass through a band pass filter, which make a semi-logarithmic scale that is easier to interpret for untrained caregivers because pattern recognition could be performed more easily compared with conventional EEG. In contrast, the accuracy and sensitivity of aEEG are a challenging topic (6). Many infrequent, low amplitude brief seizures might be missed by using EEG (12). On the other hand, focal seizures that are beyond the surface and are covered by an EEG with electrode are not detected (2). 

There is no consensus among neonatologists and neurologists regarding the treatment for clinical/electrographic seizures, the first drug of choice, and the use of EEG to diagnose and determine the treatment duration after cessation of seizures. It is controversial whether neonatal seizures, without considering underlying injury, lead to brain damage (5). It is not widely accepted that treatment of electrographic seizures per se is followed by better prognosis (13-16). Furthermore, results of basic science studies have revealed that treatment of electrical seizures leads to better prognosis, and some clinical surveys have confirmed these results (17,18). There are some evidences that MRI sequelae are also diminished by appropriate treatment (10).

No drug is recommended by FDA in neonatal seizures (19-20). However, phenobarbital is strongly suggested according to WHO guidelines, and other anti-seizure medications are used as off-label based on adult studies (21).

Phenobarbital has been used in neonatal seizures since 1912 (3). It is the most common prescribed drug in the acute phase, possibly due to tradition as well as its low cost (13, 22). By considering the time of phenobarbital prescription, many studies have been conducted to evaluate its efficacy and side effects. The results have shown that phenobarbital not only is unable to control seizure completely (13), but also could eventuate in neurocognitive consequences. Indeed, cerebral palsy is more common with the use of phenobarbital compared to other newer anti-seizure medications such as levetiracetam (23). 

The electro-clinical uncoupling phenomenon due to phenobarbital occurs in 50%-60% of cases (24) and may continue up to two years of age because of incomplete axon and myelin development (25,26). It is metabolized with the CYP2C19 enzyme and could be impressed with hypothermia or liver dysfunction that are widely observed in critically ill neonates. The most common adverse effects caused by the phenomenon are CNS and respiratory depression as well as synaptic maturation disruption and apoptosis in animal models. However, phenobarbital shows some protective features on rodents, not established in human studies (27, 28). Prescription of phenobarbital in febrile convulsion leads to lower IQ (5) and reading problems if continued after the age of two years (29). On the other hand, prenatal exposure to phenobarbital could lower verbal IQ in adulthood (30). Proapoptotic features, proven with the use of phenobarbital, are not demonstrated in animal models when using levetiracetam (31-33). 

In the recent decade, application of phenobarbital as neonatal seizure monotherapy has decreased, and levetiracetam use has become nearly 10-fold greater. Previous studies reported phenobarbital as the most common prescribed drug for monotherapy, although levetiracetam use has grown in recent years from 7% to 20%. On the other hand, combination of phenobarbital and phenytoin has been replaced by phenobarbital and levetiracetam. Thus, levetiracetam application is increasing not only in combination therapy, but also in monotherapy (1).

Levetiracetam acts through Synaptic Vesicle Glycoprotein 2A and neurotransmitters release. The mechanism of action in neonates (>37 weeks of gestation) reaches 94% of mechanism of action in adults. Thus, by knowing that the mechanism is not age-dependent, results from adult studies might be considered in neonatal seizures (34). Application in neonates shows favorable pharmacokinetics and safety profiles (35). It is used as the first choice for neonatal seizure treatment in some countries like Germany and Switzerland (21). It does not induce apoptosis, and also, has some neuro protective effects, albeit it is controversy (36, 37). 

Several surveys declare that levetiracetam not only is effective in the control of neonatal seizures (38, 39), but also has fewer side effects compared to phenobarbital (40-42, 34).

Levetiracetam does not influence cytochrome P450, and thus, it has no adverse effect on hypothermia or organ failure (43). Some of its side effects such as mild sedation, nutritional disturbances, mild apnea/bradycardia, and diminishment of urine output are considered theoretically, but are not reported in clinical cases (43, 44). Levetiracetam could be effective in neonatal seizure control up to 63%, while it is reported to have minor adverse effects (e.g., drowsiness, irritability, and transient conjugate hyperbilirubinemia) (45,9).

Table 1 briefly compares phenobarbital and levetiracetam features.

Duration of neonatal seizure treatment has not been established yet (46). Some experts believe that medications should be simplified as a sole drug before hospital discharges, or even, should be discontinued in acute seizures (47). However, others continue medications for several months despite their adverse effects (48).

Neonatal seizures are the most common neurologic manifestations in the neonatal period. In the majority of cases, there is an acute brain insult, leading to seizures such as HIE that is by far the most common etiology. It is a common issue to encourage further collaboration of neonatologists and neurologists . Since no precise definition is available for seizure diagnosis and treatment, there are much of aspects in debates. In recent years, there has been a shift from mortality to morbidity. It means that mortality has diminished from 40% to 20%, while morbidity has remained almost unchanged (i.e., about 30%) (46). As a result, we should re-evaluate our knowledge about the diagnosis and treatment of neonatal seizures. 

Recent findings suggest that new, more efficient, and less potentially harmful drugs such as levetiracetam should be used as the first line of treatment of seizures. However, there has been no convincing evidence if neonatal seizure in NICU leads to a less favorable outcome without considering the etiology (49). Moreover, it is not proven whether effective treatment of neonatal seizures yields a better neuro-cognitive outline (50).Designing a randomized clinical trial (RCT) appears to be needed for the vulnerable target population (critically ill neonates), although it is a challenging task.

In conclusion, due to the lack of adequate data and the dearth of research, use of drugs in neonatal seizure treatment should be considered from the ethical point of view. Here, we suggested a simple step by step chart to evaluate neonatal seizure, as shown in Algorithm 1.

Authors Contribution: MM designed and supervised all the processes, and ZR collected the data and prepared the primary manuscript.

**Table 1:** The comparison between phenobarbital and levetiracetam in neonatal seizure treatment

	First launch	Mechanism of action	Interaction with other drugs	Specific consideration in organ failure	Use during recent decade	Use as monotherapy	Apoptotic feature	Cost
Phenobarbital	1912	CYP2C19	Yes	yes	Decreased	Decreased	Proven	Cheaper
Levetiracetam	1999	SV2A	No	Not necessary	Increased	Increased	In doubt	More expensive

**Algorithm 1:**
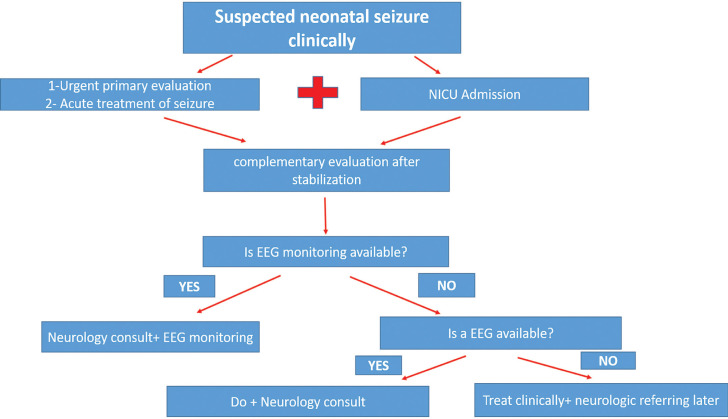
A schematic simple chart to evaluate neonatal seizure

Emergent evaluation: check for blood glucose, electrolyte, and appropriate antibiotic therapy

Complementary evaluation: brain sonogeraphy, lumbar puncture, advanced metabolic tests

## Authors Contribution

MM designed and supervised all the processes, and ZR collected the data and prepared the primary manuscript.
